# Direct Studies on the Lithium-Storage Mechanism of Molybdenum Disulfide

**DOI:** 10.1038/s41598-017-07648-0

**Published:** 2017-08-04

**Authors:** Qingmei Su, Shixin Wang, Miao Feng, Gaohui Du, Bingshe Xu

**Affiliations:** 1 Institute of Atomic and Molecular Science, Shaanxi University of Science and Technology, Xi’an, 710021 China; 2 Zhejiang Provincial Key Laboratory of Solid State Optoelectronic Devices, Zhejiang Normal University, Jinhua, 321004 China; 3 Institute of Physical Chemistry, Zhejiang Normal University, Jinhua, 321004 China; 4 Research Centre of Advanced Materials Science and Technology, Taiyuan University of Technology, Taiyuan, 030024 China

## Abstract

Transition metal sulfides are regarded as a type of high-performance anode materials for lithium ion batteries (LIBs). However, their electrochemical process and lithium-storage mechanism are complicated and remain controversial. This work is intended to give the direct observation on the electrochemical behavior and find out the lithium-storage mechanism of molybdenum disulfide (MoS_2_) using *in situ* transmission electron microscopy (TEM). We find that single-crystalline MoS_2_ nanosheets convert to Mo nanograins (~2 nm) embedded in Li_2_S matrix after the first full lithiation. After the delithiation, the Mo nanograins and Li_2_S transform to a large number of lamellar MoS_2_ nanocrystals. The discharge-charge cycling of MoS_2_ in LIBs is found to be a fully reversible conversion between MoS_2_ and Mo/Li_2_S rather than the electrochemical conversion between S and Li_2_S proposed by many researchers. The *in situ* real-time characterization results give direct evidence and profound insights into the lithium-storage mechanism of MoS_2_ as anode in LIBs.

## Introduction

The widespread application of lithium ion batteries (LIBs) promotes extensive studies on high-performance electrode materials^1, 2^. Graphite materials are generally used as anodes in commercial LIBs owing to their low cost, low working potential, and structural stability. However, the relatively low capacity (about 372 mAh g^−1^) of graphite materials cannot meet the requirement of large-scale LIBs in the future^3, 4^. To solve the problem, great efforts have been made to find promising anode materials to replace graphite^5–7^. As a typical layered transition metal sulfide, MoS_2_ has a layered structures consisting of covalently bound S-Mo-S trilayers; the MoS_2_ layers are bonded by a relatively weak van der Waals force. These structural features make MoS_2_ suitable for the intercalation of lithium ions^8^. MoS_2_ is a promising anode material because its theoretical capacity (670 mAh g^−1^ assuming 4 moles of Li^+^ insertion) can be three and a half times that of commercial graphite anodes (372 mAh g^−1^)^9–11^. Besides, compared to other emerging anode materials (like Ge and Si), MoS_2_ generally demonstrates high capacity retention and excellent rate capability. Especially, nanostructured MoS_2_-based anodes have been extensively studied and their electrochemical performances have been further improved^12–17^.

Researches on the reaction mechanism of MoS_2_ as anode in LIBs are significant for both fundamental studies and practical applications. The intercalation of lithium ions into MoS_2_ happens in the voltage range of 3.0–0 V with varied reaction mechanisms. Lithium intercalation is believed to be reversible in the voltage of 3–1.1 V via the reaction: MoS_2_ + *x*Li^+^  + *x*e^−^ ↔ Li_*x*_MoS_2_. At voltages below 1.1 V there exist one or several electrochemical reactions along with the formation of intermediate metastable sulfides. The complex mechanism of lithium intercalation into MoS_2_ under deep discharge has been preliminarily investigated. Different reaction mechanisms for the conversion process of MoS_2_ with Li have been recently proposed by various groups. Lemmon and co-workers reported that MoS_2_ was reduced to metallic Mo and Li_2_S at 0.01 V (*vs*. Li/Li^+^)^18^. Wang and Li believed that the lithium storage mechanism of MoS_2_ is a reversible phase transformation between MoS_2_ and Mo/Li_2_S^19^, and major researches agree with this reversible conversion mechanism^20–25^. However, many groups suggested that the conversion mechanism of MoS_2_ is analogous with Li–S battery due to the formation of Li_2_S during the first discharge process according to the redox chemistry^12, 26–28^; the subsequent reaction is a reversible conversion between Li_2_S and elemental sulfur: Li_2_S ↔ S + 2Li. It says metallic Mo is not active and Li_2_S/S is the electrochemical reaction couple in a deeply discharged MoS_2_/Li cell. Thus, the electrochemical reaction between MoS_2_ and Li needs thorough enlightenment owing to these controversial results.

Considerable understandings of MoS_2_ were achieved by examining the electrode materials after charge or discharge by disassembling the conventional LIBs (i.e. *ex situ*); no report provided direct reaction behavior of MoS_2_ electrode during the discharge-charge cycling to analyze the lithium-storage mechanism. *In situ* TEM is an advanced technique that allows specific nanoscale site on the materials to be monitored in real-time^29–37^. The lithium intercalation into MoS_2_ nanosheet has been reported by Bai’s group through *in situ* TEM approach^36^, and they demonstrated a phase conversion from pristine 2H-MoS_2_ to 1T-LiMoS_2_. Their finding confirms the initial Li^+^ intercalation mechanism into MoS_2_. The controversial conversion mechanism involving the deep discharge of the battery (at approximate 0 V *vs*. Li/Li^+^) is not resolved. Particularly, the microstructural evolution of MoS_2_ electrode during continuous discharge-charge cycling remains unknown.

In this work, we investigated the lithium intercalation and conversion process of lamellar MoS_2_ nanosheets in deep cycle (like the battery operation between 0–3 V) by *in situ* TEM, and realized a real-time observation of the electrochemical lithiation and delithiation process that greatly advances the understanding of the intercalation and conversion mechanism of MoS_2_ in LIBs. The experimental results, for the first time, thoroughly demonstrate the structural transition of MoS_2_ is reversible in the full discharge-charge cycling. Our findings shed light on the electrochemical reaction of MoS_2_ with lithium and benefit for future material design and applications in energy conversion and storage devices.

## Results

**Figure 1:**
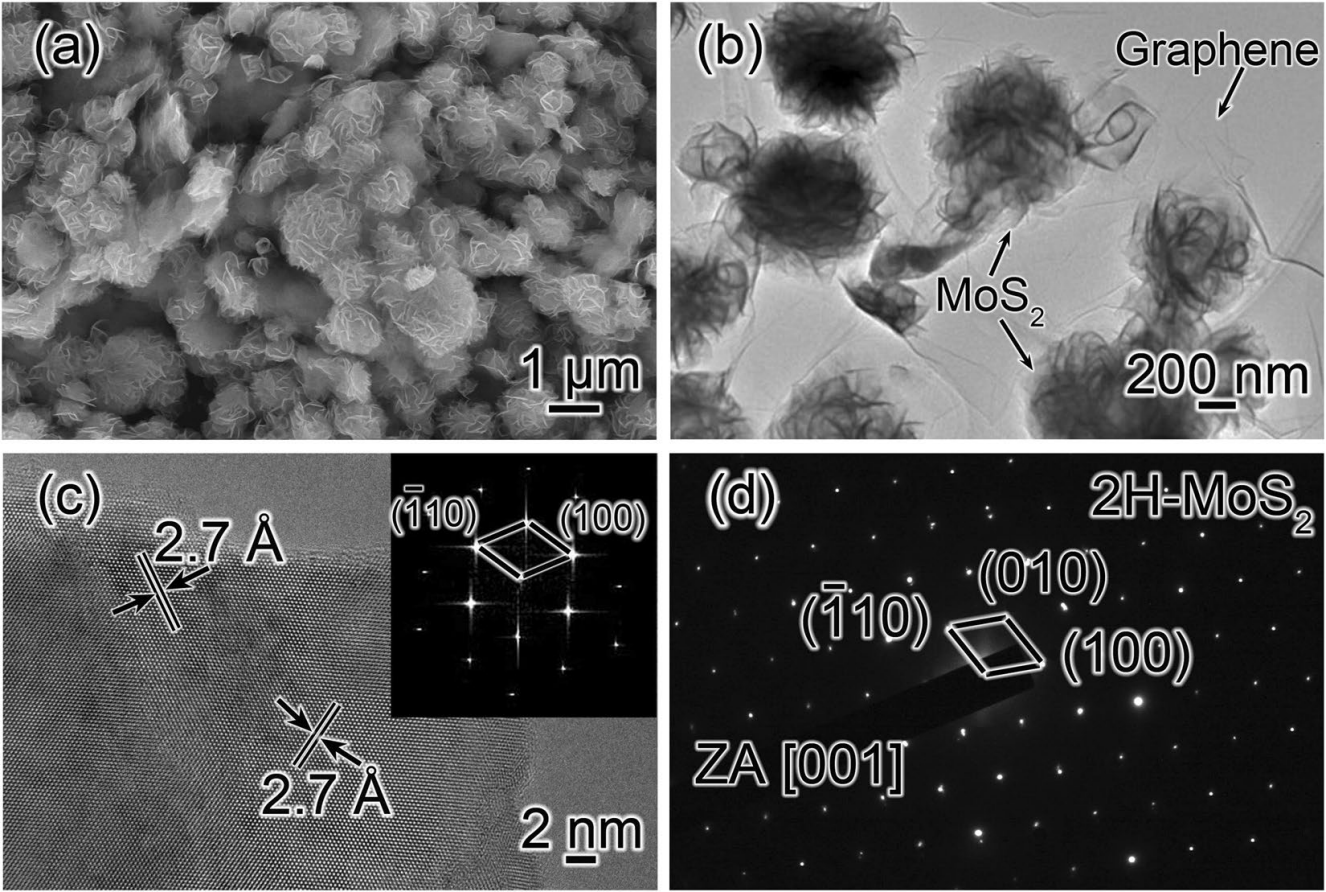
(**a**) SEM and (**b**) TEM images of the MoS_2_/graphene products. (**c**) HRTEM image and (**d**) SAED pattern of an individual MoS_2_ nanosheet. The inset of (**c**) is the corresponding FFT pattern of the HRTEM image.

The morphology and microstructure of the synthesized MoS_2_/graphene were analyzed by SEM and TEM. Figure 1(a) presents a SEM image of the obtained MoS_2_/graphene used in this study; the MoS_2_/graphene is composed of many uniform MoS_2_ microspheres with sizes of 1–2 μm. Figure 1(b) clearly reveals the microstructures of the MoS_2_ microspheres anchored on graphene nanosheet. It can be seen that the hierarchical flower-like microspheres are regularly composed of numerous thin nanosheets. Figure 1(c) shows a HRTEM image of an individual MoS_2_ sheet in the microspheres. The marked interplanar spacing of 2.7 Å corresponds to the (100) and ($\bar{1}$10) lattice planes of the hexagonal MoS_2_ phase, respectively. The inset is the corresponding fast Fourier transform (FFT) pattern of the HRTEM image. The SAED pattern recorded from an individual MoS_2_ nanosheet shows clear diffraction spots and can be well indexed as (100), (010) and ($\bar{1}$10) planes of pure hexagonal MoS_2_ along the[001] zone axis (JCPDF No. 87-2416), indicating a high crystallinity of MoS_2_ phase.

**Figure 2:**
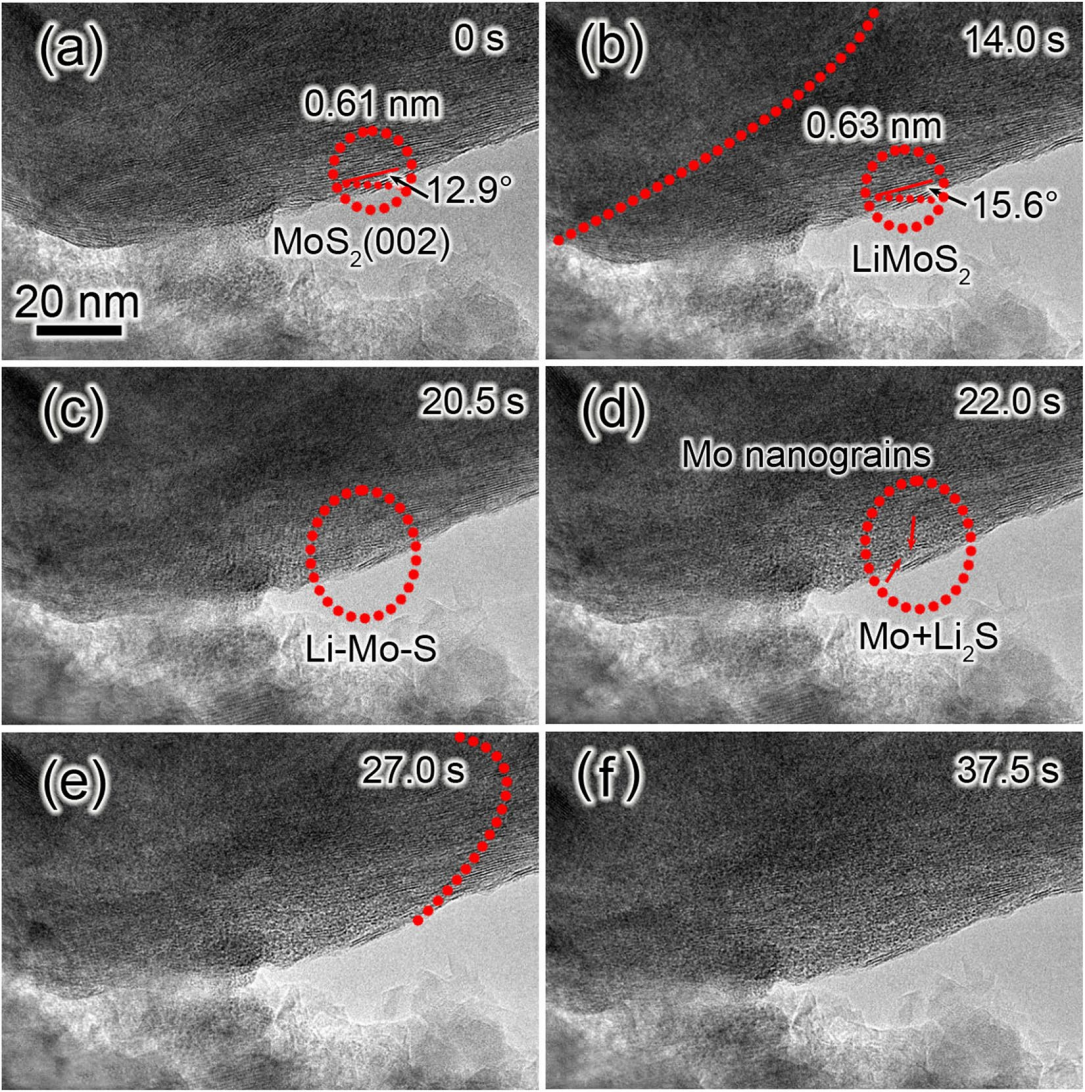
*In situ* TEM study on the lithiation of an individual MoS_2_ nanosheet. (**a**–**f**) Time-resolved TEM images of the first lithiation process. All the scale bars are 10 nm.

We prepared an all-solid nanoscale LIB inside the TEM that enabled the *in situ* electrochemical experiments of MoS_2_/graphene. Graphene nanosheets in the composite were used as substrate to support the MoS_2_ nanostructures so that the *in situ* observation could be easily performed. The nanosized electrochemical cell consisted of three parts: MoS_2_/graphene working electrode, Li metal counter electrode, and naturally grown Li_2_O on Li metal surface used as solid electrolyte. The TEM images of Li/Li_2_O electrode are shown in Fig. S1 in Supporting Information. ED pattern and elemental mapping analysis confirm the existence of Li_2_O layer on Li metal. So the MoS_2_/graphene electrode is not directly contacted with lithium metals; the observed reaction is an electrochemical process rather than electrical short-circuit. Subsequently, the electrochemical conversion reaction of MoS_2_ nanosheets during the first lithiation process was investigated. Figure 2 shows a series of time-resolved TEM images of a MoS_2_ nanosheet during the first lithiation, which is a screenshot from Movie S1 in Supporting Information. The marked interplanar spacing of 0.61 nm corresponds to the (002) lattice plane of the hexagonal MoS_2_ phase. Figure 2(a) was used as the starting point to monitor the progress of the lithiation reaction. Three stages in the first lithiation process can be discerned from Movie S1 in the Supporting Information. The first stage, from 0 to 14.0 s, indicates the initial Li^+^ intercalating reaction with MoS_2_. We can see the slight variation of the orientation of lattice fringes in the TEM images and Movie S1 due to the strain induced by the lithium intercalation, leading to the formation of Li_x_MoS_2_ phase via 2H-1T transformation^36^. The lattice fringes of 0.63 nm in Fig. 2(b) corresponds to the (001) lattice plane of the LiMoS_2_ phase (JCPDF No. 44-1078). The images recorded from 14.0 s to 27 s are the second stage (Fig. 2b–e), in which the crystalline MoS_2_ layers began to collapse, leading to the formation of small Mo-Li-S clusters and then Mo clusters and Li_2_S phase. In Fig. 2(c–e), many nanodots resulted from the decomposition of a MoS_2_ monolayer should be Mo clusters. In the stage three, Mo clusters aggregated and grew into Mo nanograins as shown in Fig. 2(f). The continuous lithiation was revealed by the change of the texture of MoS_2_ nanosheet in the TEM images. Most of the lattice fringes of MoS_2_ nanosheet disappeared and numerous ultrafine nanograins were formed owing to lithiation reaction in Fig. 2(f).

**Figure 3:**
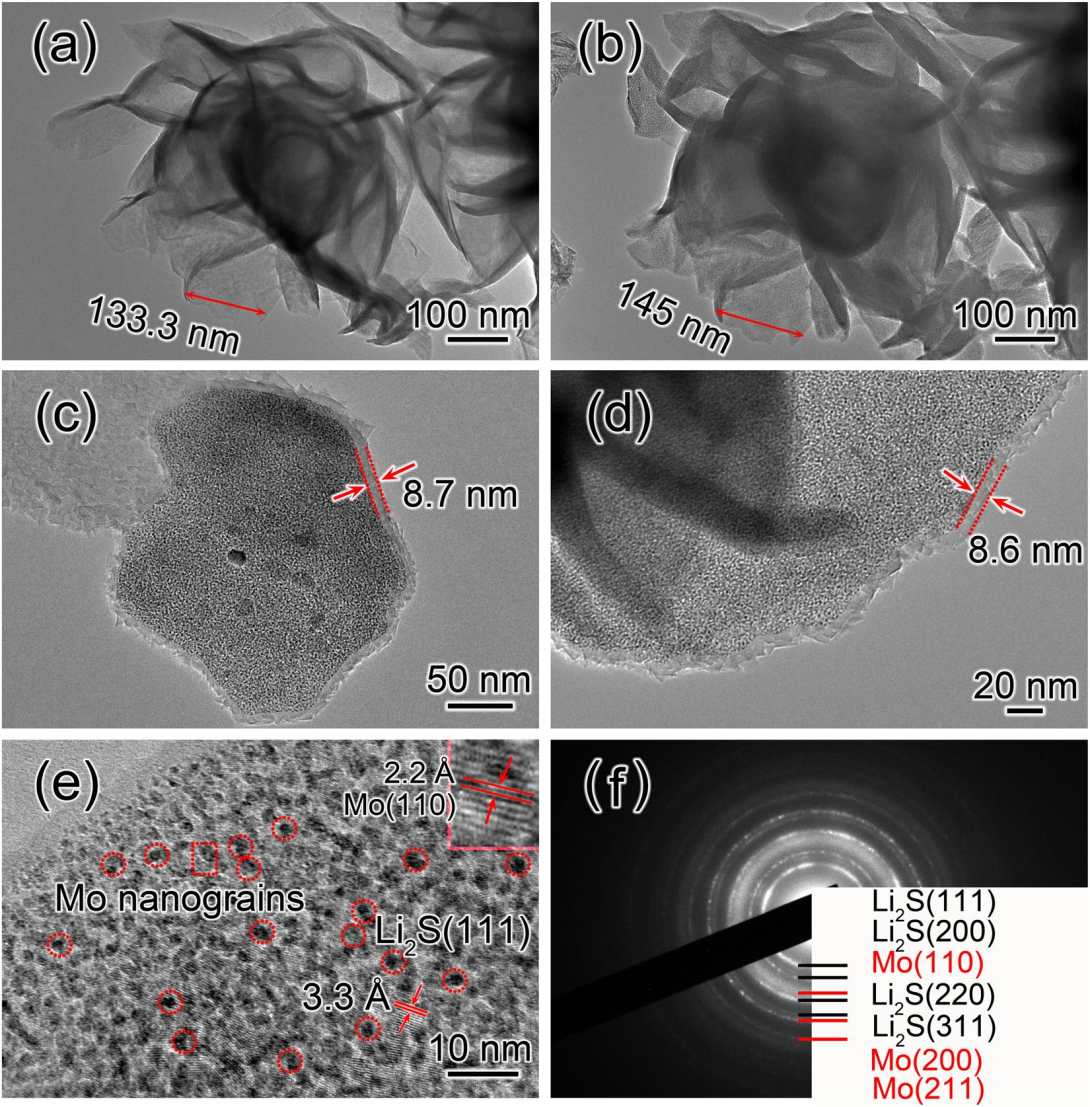
(**a**) TEM image of a pristine MoS_2_ microsphere before lithiation. (**b**) TEM image of MoS_2_ microsphere after first lithiation. (**c**,**d**) TEM images of the lithiated MoS_2_ nanoflakes selected from the MoS_2_ electrode in (**b**). (**e**) HRTEM image and (**f**) ED pattern of the fully lithiated MoS_2_ microsphere in (**b**).

Figure 3(a) is a TEM image of a pristine MoS_2_ microsphere; the initial size of the marked MoS_2_ nanosheet was 133 nm. After the first full lithiation, the size of MoS_2_ nanosheet increased to 145 nm as shown in Fig. 3(b), and the corresponding size expansion was ~8.8%. The TEM images of an individual MoS_2_ nanoflake after the first lithiation are shown in Fig. 3(c,d). The surface and edges of the lithiated MoS_2_ nanoflake were coated by a uniform layer of crystallite with a thickness of 8–9 nm, which was identified to be Li_2_S from the ED pattern in Fig. 3(f). Figure 3(e) shows the detailed structure information of the lithiated MoS_2_ nanoflake, which is composed of numerous nanograins around 2 nm. The HRTEM image of a nanograin in the lithiated MoS_2_ nanoflake gives the lattice fringes of 2.2 Å (the inset of Fig. 3e), which is agreed with the lattice spacing of (110) plane of Mo (JCPDF No. 89-5156). The fringe spacing of the matrix was measured to be 3.3 Å, corresponding to the (111) plane of Li_2_S (77–2145). The dense Mo nanograins form an interconnected network that works as an efficient conductive pathway for electron transport into the MoS_2_, while the Li_2_S provides a similar pathway for Li ions during electrochemical reaction. Since Li_2_S has a much smaller density (1.66 g/cm^3^) than that of metal Mo (10.28 g/cm^3^), the volumetric expansion of MoS_2_ electrode during the lithiation process is mainly caused by the formation of Li_2_S with lower density. Li_2_S is soft and the resulted Li_2_S in the nanoflakes could be partially squeezed out of the Mo network/framework due to volumetric expansion. So Li_2_S shells with a thickness of 8–10 nm were formed around the fully lithiated nanoflakes. Figure 3(f) is the ED pattern of the fully lithiated MoS_2_ electrode. All the diffraction rings are well indexed into Mo and Li_2_S. The results demonstrate the lithiation reaction involves the reduction of MoS_2_ to Mo nanograins accompanying with the formation of Li_2_S. The whole lithiation reaction can be expressed as the following equation: MoS_2_ + 4Li^+^  + 4e^−^ → Mo + 2Li_2_S.

**Figure 4:**
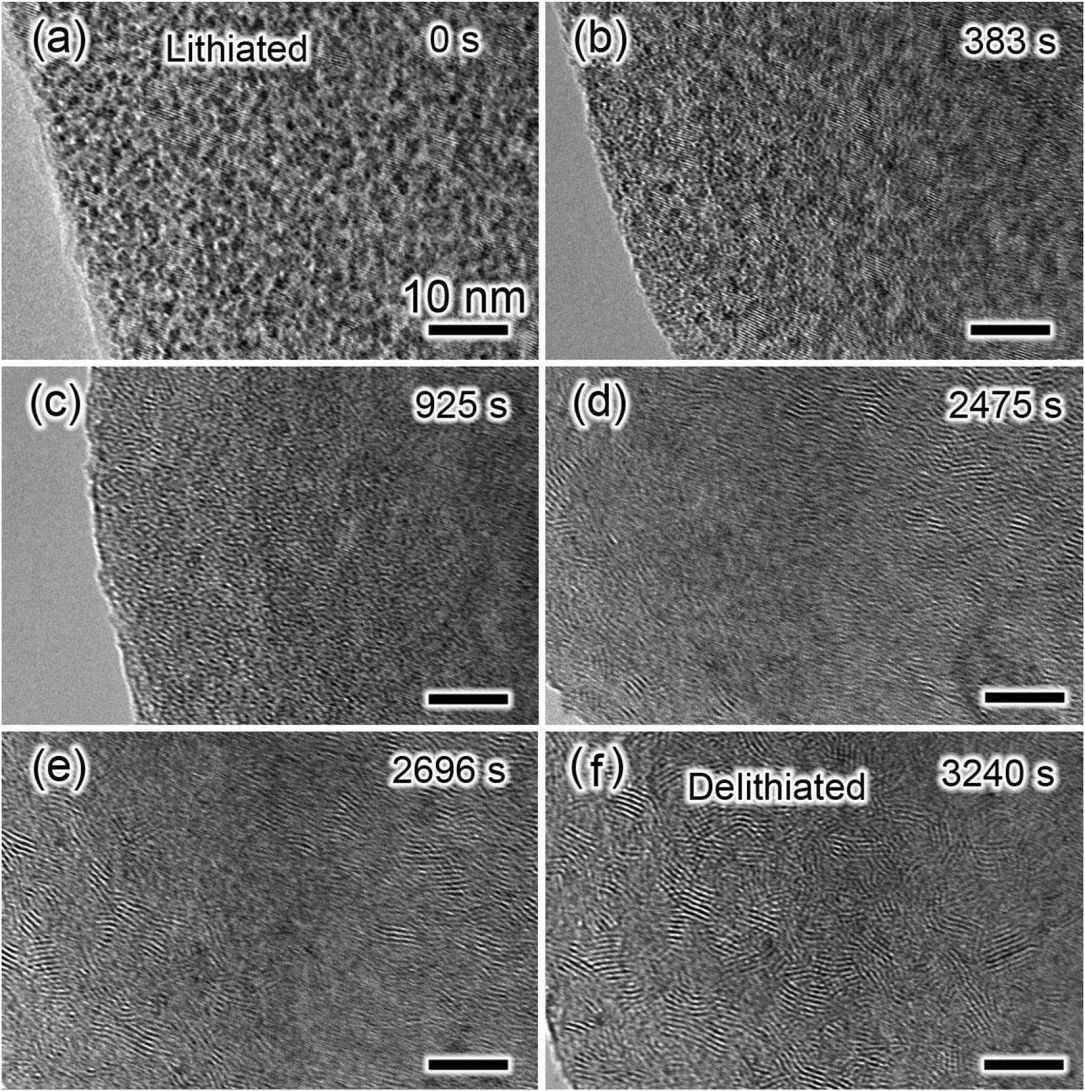
Time-resolved TEM images of a lithiated MoS_2_ nanosheet during the delithiation process showing obvious microstructure and phase evolution as a function of time. (**a**) 0 s, (**b**) 383 s, (**c**) 925 s, (**d**) 2475 s, (**e**) 2696 s and (**f**) 3240 s. All the scale bars are 10 nm.

After the first lithiation process, a potential of +3.0 V was applied to the lithiated MoS_2_ electrode to initiate the delithiation reaction. The time-resolved TEM images of the electrode during the delithiation are shown in Fig. 4. Figure 4(a) is a TEM image of the lithiated MoS_2_ electrode, in which numerous Mo nanograins (black nanodots) around 2 nm are embedded in the Li_2_S matrix. After the delithiation reaction for 383 s (Fig. 4b), an obvious change could be observed in the nanosheet electrode in comparison to the image in Fig. 4a. The Mo nanograins became smaller due to the electrochemical reaction with Li_2_S during delithiation. As the delithiation reaction proceeded, black nanodots involving Mo nanograins almost disappeared, and the electrode turned to an approximately amorphous texture after 925 s (Fig. 4c). Obvious formation of lattice fringes in the electrode was observed at 2475 s during the delithiation process (Fig. 4d). After reaction for 3240 s (Fig. 4f), we found the perfect lattice fringes were formed, indicating the completion of delithiation reaction.

**Figure 5:**
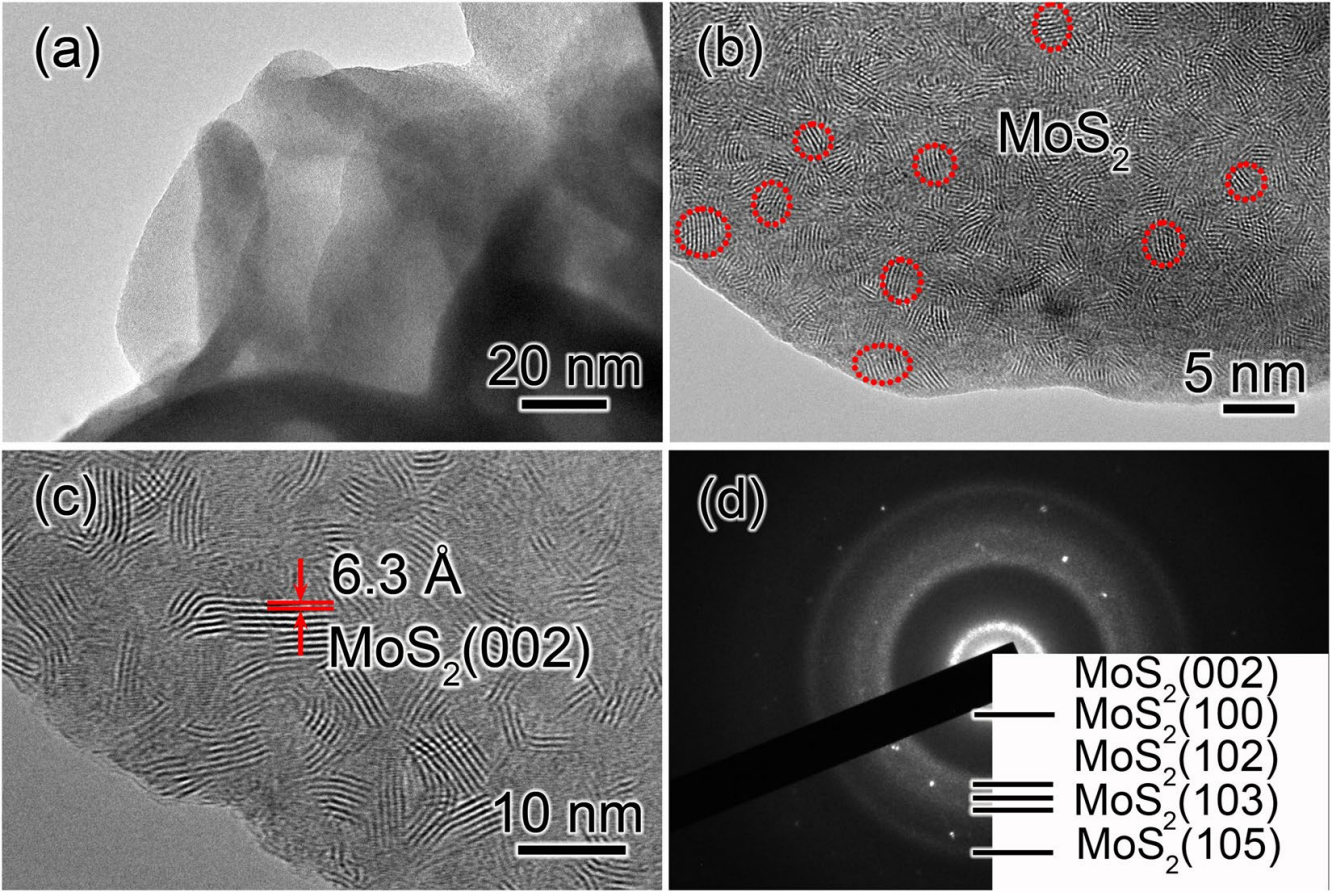
(**a**) TEM image of MoS_2_ electrode after delithiation with a potential of +3.0 V. (**b**) The enlarged TEM image of a delithiated MoS_2_ sheet. (**c**) HRTEM image and (**d**) ED pattern of a delithiated MoS_2_ nanosheet.

The delithiated MoS_2_ electrode was analyzed using TEM. Figure 5(a) is a TEM image of the delithiated MoS_2_ electrode. It can be clearly seen that the thin Li_2_S shell on lithiated nanosheets was disappeared. The magnified TEM image of the delithiated MoS_2_ nanosheet is displayed in Fig. 5(b), suggesting that a large number of lamellar nanocrystals with a size of 3–5 nm formed during the delithiation process, as marked by the red circles. Figure 5(c) is a HRTEM image of the delithiated MoS_2_ nanosheet; the interplanar distance of nanograins was measured to be 6.1 Å, which can be indexed as the (002) lattice planes of the hexagonal MoS_2_ phase (JCPDF No. 87-2416). The ED pattern of the delithiated nanosheet is shown in Fig. 5(d); all the diffraction rings can be indexed as the hexagonal MoS_2_ phase (JCPDF No. 87-2416). The HRTEM and ED results reveal that metallic Mo nanograins convert to MoS_2_ nanograins in the delithiation process. Here molybdenum oxide was not observed during the delithiation process although Li_2_O electrolyte was contacted with the electrode. The electrochemical reaction in the delithiation process can be expressed as Mo + 2Li_2_S → MoS_2_ + 4Li^+^  + 4e^−^.

**Figure 6:**
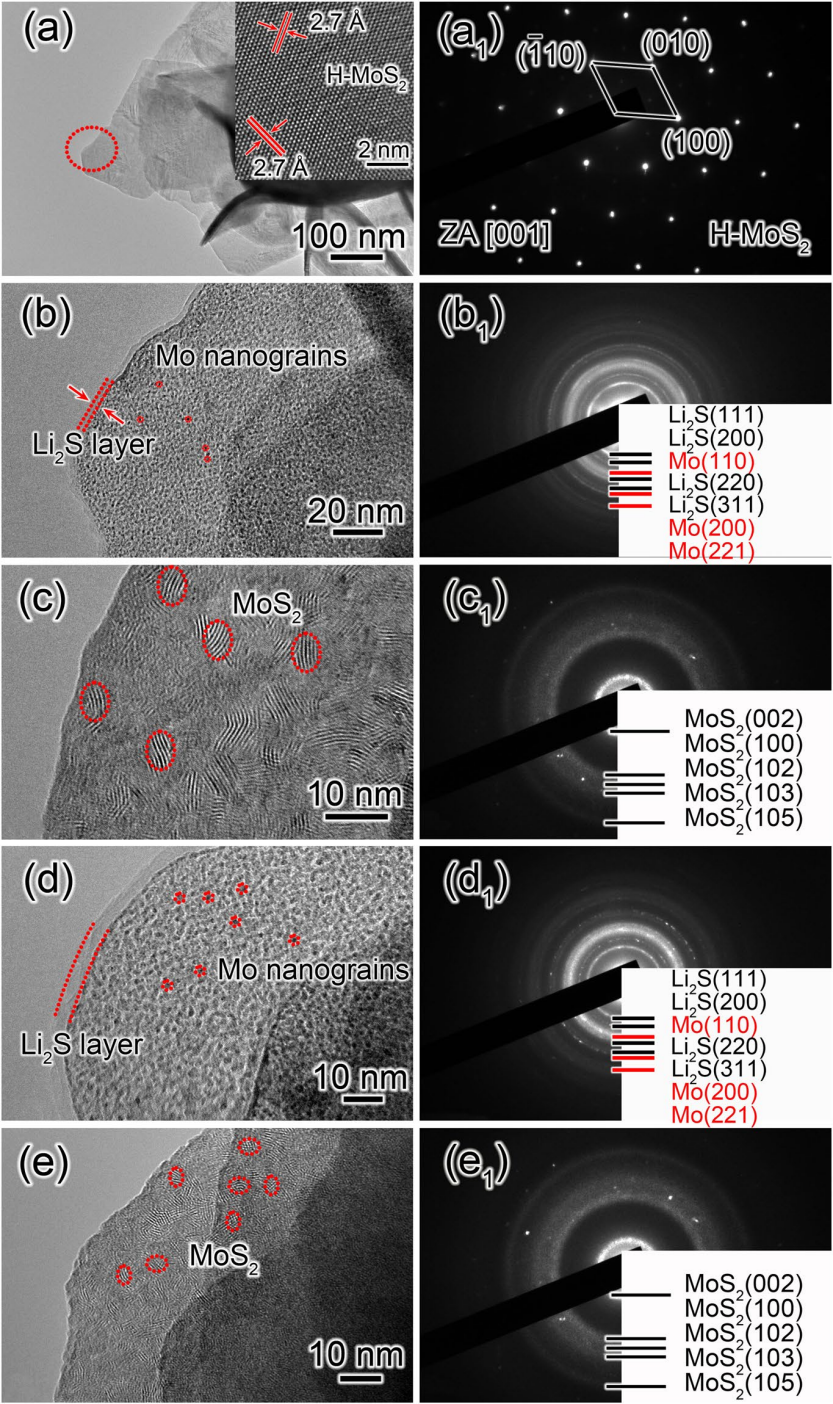
Morphology and structure evolution of MoS_2_ electrode during cycling with the potential of −1.0 V for lithiation and +3.0 V for delithiation. (**a**) TEM image of the pristine MoS_2_ nanosheet. (**b**,**c**) TEM images of MoS_2_ nanosheet after the first lithiation and delithiation. (**d**,**e**) TEM images of MoS_2_ nanosheet after the second lithiation and delithiation. (**a**_**1**_–**e**_**1**_) The corresponding ED patterns of the MoS_2_ electrode in (**a**–**e**).

MoS_2_ materials showed excellent cyclability during the charge-discharge cycles in LIBs^12–14^. *In situ* TEM experiments were performed to reveal the microstructure and phase evolution induced by the continual lithiation-delithiation in the discharge-charge cycles. Figure 6(a) is a TEM image of the pristine MoS_2_ electrode, and the insets is a HRTEM image of the MoS_2_ nanosheet. The marked interplanar spacing of 2.7 Å corresponds to the (100) and ($\bar{1}$10) lattice planes of the hexagonal MoS_2_ phase, respectively. The ED pattern of the pristine MoS_2_ nanosheet is displayed in Fig. 6(a_1_). It can be indexed as (100), (010) and ($\bar{1}$10) planes of pure hexagonal MoS_2_ phase along the[001] zone axis (JCPDF No. 87-2416). Figure 6(b) is a TEM image of MoS_2_ nanosheet after the first lithiation, and a thin Li_2_S shell with a thickness of 8–9 nm was formed on the lithiated nanosheet. Meanwhile numerous Mo nanograins (~2 nm) were formed and embedded in Li_2_S matrix during the lithiation reaction. The corresponding ED pattern of the lithiated MoS_2_ electrode is shown in Fig. 6(b_1_). The diffraction rings can be well indexed to Mo and Li_2_S, suggesting the conversion of MoS_2_ to Mo and Li_2_S in the first lithiation process. The potential was reversed to +3 V to initiate the delithiation process after the completed lithiation. Figure 6(c) is the TEM image of the delithiated MoS_2_ electrode. It shows that the thin Li_2_S shell disappeared. The Mo nanograins transformed into a large number of MoS_2_ nanograins with sizes of 3–5 nm during the delithiation reaction, as marked by the red circles. Figure 6(c_1_) displays the ED pattern of the delithiated MoS_2_ electrode; the diffraction rings all originate from lattice planes of the hexagonal MoS_2_ phase (JCPDF No. 87-2416). The second lithiation process proceeded with the potential of −1.0 V again as shown in Fig. 6(d). Movie S2 in the Supporting Information also reveals the dynamic conversion of the electrode material in the second lithiation process. The Li_2_S layer formed and continued to cover the electrode like the first lithiation. The ED pattern of the secondly lithiated electrode is shown in Fig. 6(d_1_) and can be indexed as Mo and Li_2_S. The Li_2_S layer and Mo nanograins disappeared again in the second delithiation process (Fig. 6e), showing a reversible phase change. The ED pattern in Fig. 6(e_1_) indicated the resultant Mo/Li_2_S converted to MoS_2_ nanograins again in the second delithiation process. The two cycles indicate that a reversible electrochemical conversion between MoS_2_ and Mo/Li_2_S takes place in the whole cycling, and the electrochemical reaction in the discharge-charge cycling can be expressed as MoS_2_ + 4Li^+^  + 4e^−^ ↔ Mo + 2Li_2_S.

The electrochemical performances of MoS_2_/graphene electrode in coin cells were tested. As shown in Fig. S2, the initial discharge and charge capacities are 2018 mAh g^−1^ and 1609 mAh g^−1^, respectively. After 100 cycles, the reversible capacity of the MoS_2_/graphene electrode remains 1002 mAh g^−1^, demonstrating a high lithium–storage capability and excellent cycling performance. The reversible capacity is higher than the theoretic capacity of MoS_2_, which can be attributed to the synergistic effects of graphene and the reversible formation/decomposition of SEI membrane.

The *in situ* TEM results were further supported by *ex situ* TEM results, as displayed in Fig. S3 in Supporting Information. The TEM image of the MoS_2_/graphene electrode after 50 discharge-charge cycles shows that numerous ultrafine nanograins were formed (Fig. S3(a)). The stripe-width of the crystallites is 2.2 Å, which corresponds to the interplanar spacing of Mo (110) plane (Fig. S3(b)). The HRTEM analysis confirms the formation of Mo nanograins after discharge. Meanwhile, Fig. R3(c) and (d) indicate that the MoS_2_ phase was obtained after charge. The *ex situ* TEM results are well agreed with our *in situ* TEM results, and confirms the same conversion mechanism revealed by *in situ* TEM. The findings clarified the lithium-storage mechanism of MoS_2_ involving the reversible phase transformation between Mo nanograins and MoS_2_ nanograins along with the reversible growth/decomposition of Li_2_S during cycling. The reactions are analogous to the electrochemical conversion of CoS_2_^38^, and most transition-metal oxides^39^. Thus, the electrochemically active couple in MoS_2_ electrode is not S/Li_2_S but MoS_2_ and Mo/Li_2_S in a deeply discharged/charged MoS_2_/Li system.

Our *in situ* and *ex situ* TEM results reveal a reversible phase conversion during the lithitation-delithiaton processes of MoS_2_. However, the typical CV curves of MoS_2_ in literatures generally show distinct 1st and subsequent discharge processes^40–43^. The difference in the CV curves may be caused by the irreversible processes such as the decomposition of electrolyte in the first discharge^40^, the formation of a solid-electrolyte interface (SEI) layer resulting from the continual reaction of freshly exposed surfaces of electrode materials with the organic electrolyte^41^, and particularly the structural rearrangement revealed by our TEM observation in the first discharge-charge process from micro-sized MoS_2_ nanosheets to numerous MoS_2_ nanograins of 3–5 nm, which possess distinct quantum and size effects. Besides, the MoS_2_ nanostructures prepared by the hydrothermal or solution method contain many defect sites. A fraction of lithium ions can be trapped in the defect sites and hardly extracted in the following cycles^42^. The residual oxygen-containing groups in graphene for MoS_2_/graphene composite are also electrochemical reduced in the first discharge^43^. All these irreversible processes can cause different reaction thermodynamics between the 1st and subsequent discharge. The underlying mechanism still needs thorough investigation.

## Conclusions

In summary, we have conducted a systematical study of the structural evolution of MoS_2_ nanosheets during the lithiation-delithiation cycling using *in situ* TEM technique by constructing a nano-LIB device inside a TEM. In our experiments, the details of Li-ion intercalation and extraction-induced solid-state phase transformation in MoS_2_ are clearly understood. The results demonstrate that single-crystalline MoS_2_ nanosheets transform to multicrystalline nanosheets consisting of many Mo nanograins embedded in Li_2_S matrix during the first lithiation. Generally, a uniform layer of Li_2_S with thickness of 8–9 nm can be observed on the lithiated electrode. During the delithiation process, Mo nanograins and Li_2_S layer convert to a large number of lamellar MoS_2_ nanograins with sizes of 3–5 nm. The charge-discharge processes of MoS_2_ in LIBs are not the controversial electrochemical conversion between S and Li_2_S but a fully reversible phase conversion between MoS_2_ nanograins and Mo nanograins along with the formation/decomposition of Li_2_S. Based on our *in situ* TEM results, the electrochemical conversion mechanism of MoS_2_ in LIBs is: MoS_2_ + 4Li^+^  + 4e^−^ ↔ Mo + 2Li_2_S. The *in situ* real-time characterization results provide direct evidence and achieve a profound understanding of the lithium-storage mechanism in MoS_2_.

## Methods

### Materials synthesis

Graphene Oxide (GO) was prepared by oxidization of natural graphite flakes using a modified Hummers method^44^. For the synthesis of MoS_2_/graphene, GO (0.06 g) was added into 20 mL of deionized water with sonication for 1 h to form a homogeneous dispersion. Then 0.3 g of Na_2_MoO_4_·2H_2_O, 0.4 g of thiourea, and 0.15 g of PEG-20000 were added. After ultrasonication and stirring for 30 min, the solution was then transferred into a Teflon-lined stainless steel autoclave, sealed tightly, and heated at 200 °C for 24 h. The black products were collected by centrifugation, washed with deionized water and ethanol, and dried in a vacuum oven at 60 °C for 24 h. The obtained composites were annealed in a tube furnace at 800 °C for 2 h in a stream of hydrogen (20 sccm) and argon (180 sccm).

### *In situ* electrochemical experiments inside TEM

The *in situ* nanoscale electrochemical experiments were performed using a Nanofactory STM-TEM holder inside a TEM (JEOL JEM-2100F). MoS_2_/graphene samples were attached to a gold wire and served as the working electrode; a lithium particle on the tip of a W rod was served as the counter electrode and lithium source. Both the MoS_2_/graphene and lithium electrodes were loaded onto a Nanofactory STM-TEM holder inside a glovebox and sealed in an argon-filled bag. Then the holder was transferred into the TEM column quickly. A thin Li_2_O layer was formed on the surface of the metallic lithium particle due to the exposure to air during the transfer process and was served as the solid electrolyte. Once the Li_2_O-covered Li electrode driven by the mobile STM probe contacted the free end of the selected lamellar MoS_2_/graphene, a negative bias (−1.0 V) was applied to drive Li^+^ transport through the solid-state Li_2_O layer to facilitate the lithiation. The current of about tens or hundreds nA passed through the sample during the reaction (dependent on the size of the selected sample). Then a bias was reversed to be positive (+3.0 V) to initiate the delithiation process. To minimize the electron beam irradiation effect on the microstructure and the electrochemical behavior, the experiments were carried out with the electron beam blanked except for imaging. During the image acquisition, the total dose of electron beam was reduced with sacrifice in resolution and contrast. In some cases, the beam radiation was performed in a sacrificial region and the TEM measurements were taken on a nearby region that had not been previously exposed to the electron beam.

## Electronic supplementary material

Movie S1

Movie S2

Supporting information
