# Supplementary material for: Direct Studies on the Lithium-Storage Mechanism of Molybdenum Disulfide
Source: Sci Rep. 2017 Aug 4;7:7275. doi: 10.1038/s41598-017-07648-0 (PMC5544753; doi:10.1038/s41598-017-07648-0)
Supplement: Supplementary file 3 — Supporting information [file 41598_2017_7648_MOESM3_ESM.pdf]

-Supporting Information-

Direct Studies on the Lithium-Storage Mechanism of Molybdenum Disulfide

Qingmei Su[1,2,*], Shixin Wang[1], Miao Feng[3], Gaohui Du [1,3,*], Bingshe Xu[1,4]

[1]Institute of Atomic and Molecular Science, Shaanxi University of Science and Technology, Xi'an 710021, China.

[2] Zhejiang Provincial Key Laboratory of Solid State Optoelectronic Devices, Zhejiang Normal University, Jinhua 321004, China.

[3] Institute of Physical Chemistry, Zhejiang Normal University, Jinhua 321004, China.

[4]Research Centre of Advanced Materials Science and Technology, Taiyuan University of Technology, Taiyuan 030024, China.

*Correspondence and requests for materials should be addressed to Q.S. (email: suqingmei@zjnu.cn) or G.D. (email: dugaohui@sust.edu.cn)

1. Electrochemical measurement

The MoS_2/graphene electrode was prepared by dispersing the as-prepared MoS_2/graphene (75 wt. %), acetylene carbon black (15 wt. %) and polyvinylidenefluorides (PVDF) binder (10 wt. %) in N-methyl-2-pyrrolidone (NMP) solvent to form a slurry. The mixture was spread onto Ni foam current collector and dried at 80 ℃ in a vacuum oven for 12 h. Then the as-prepared Ni foil was cut into a disk with a diameter of 12 mm as the working electrode. The electrochemical measurements were carried out by two-electrode coin cells (CR2025). The test CR2025 coin cells were assembled in an argon-filled glove box with the metallic lithium foil as the counter electrode, 1 M $LiPF_6$ in ethylene carbonate (EC)-dimethyl carbonate (DMC) (1:1 in volume) used as the electrolyte, and a polypropylene (PP) microporous film (Cellgard 2300) employed as the separator. The galvanostatic charge-discharge measurements were tested at current density of 100 mAh g^{-1} between cut-off voltages of 0.01-3 V at room temperature.

2. Supporting Movies

Movie_S1.avi

In situ TEM movie shows the electrochemical lithiation process of an individual MoS_2 nanosheet during the first cycle. The video was recorded at 4 frames/s.

Movie_S2. avi

In situ TEM movie shows the electrochemical lithiation process of an individual MoS_2 nanosheet during the second cycle. The video was recorded at 4 frames/s.

3. Supporting Figures

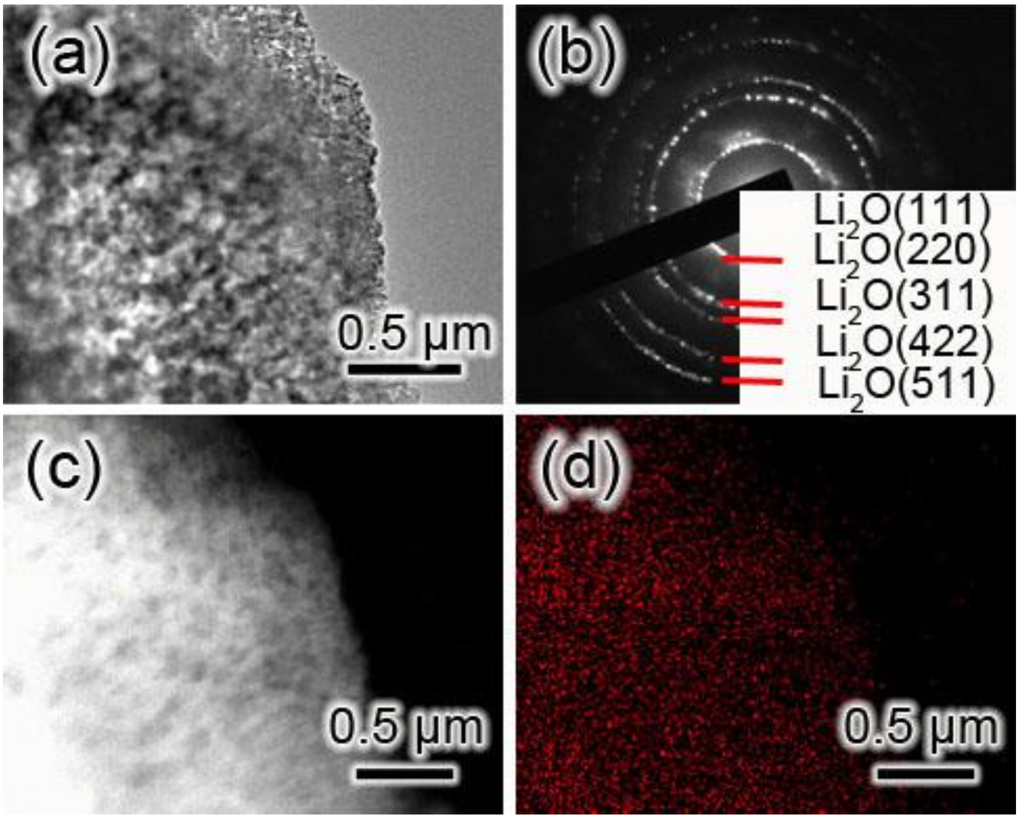

Figure S1 (a) TEM image of Li/Li_2O electrode. (b) ED pattern of Li_2O layer. (c) Dark-filed STEM image and (d) Elemental mapping of O in Li/Li_2O electrode. The ED pattern and elemental mapping image indicate that the surface of lithium metal has a thin Li_2O layer.

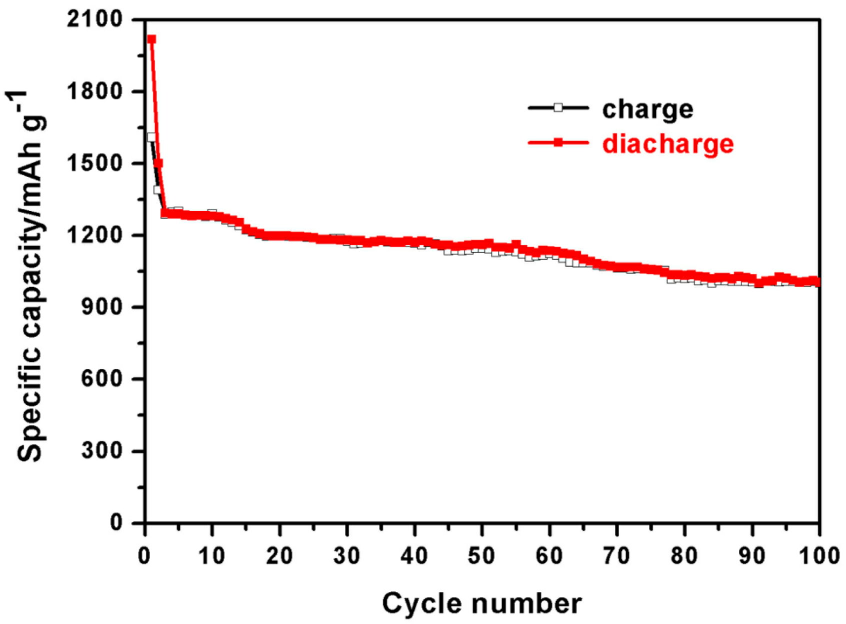

Figure S2. Reversible charge/discharge capacities against cycle number at a current density of 100 mA g^{-1}.

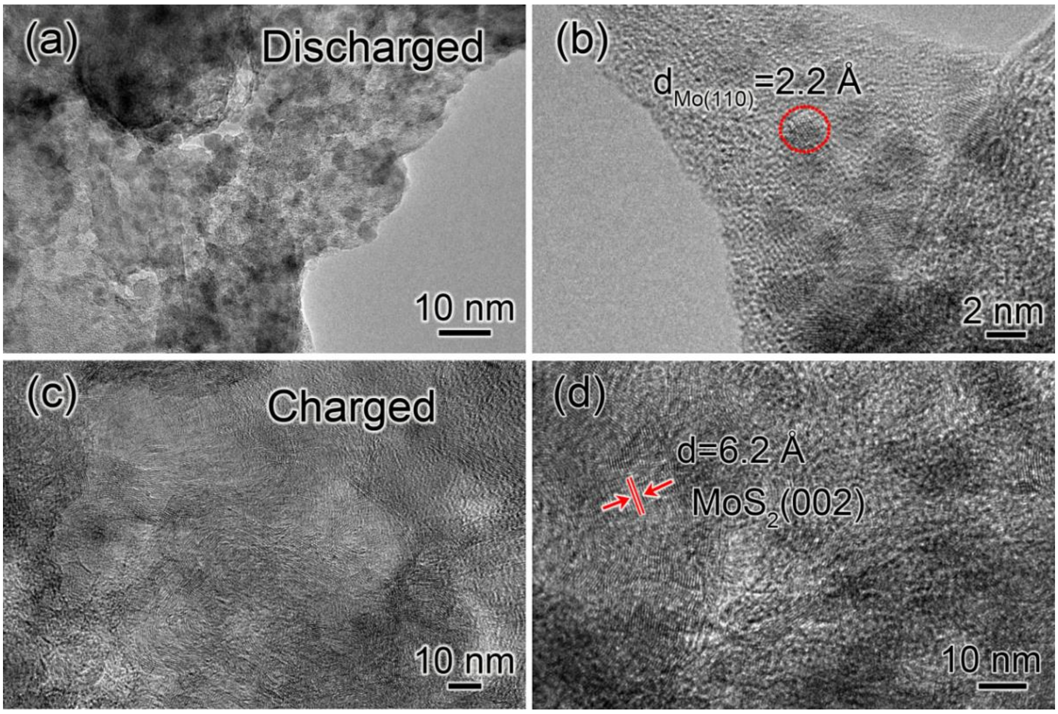

Figure S3. (a, b) TEM and HRTEM images of MoS_2/graphene electrode fully discharged to 0.01 V. (c, d) TEM and HRTEM images of MoS_2/graphene electrode fully charged to 3.00 V after 50 cycles.
